# Genomic profiling of CD20 negative diffuse large B cell lymphoma identifies targetable mutations: A case report

**DOI:** 10.1002/jha2.93

**Published:** 2020-09-04

**Authors:** M. Gohar Maqbool, Jun Hee Lim, Sanjiv Jain, Dipti Talaulikar

**Affiliations:** ^1^ Department of Haematology ACT Pathology Canberra Hospital Canberra ACT Australia; ^2^ Haematology Translational Research Unit Canberra Hospital Canberra ACT Australia; ^3^ ANU Medical School College of Medicine and Health Australian National University Canberra ACT Australia; ^4^ Department of Anatomical Pathology ACT Pathology Canberra Hospital Canberra ACT Australia

Diffuse large B cell lymphoma (DLBCL) is a clinically and genetically heterogeneous non‐Hodgkin lymphoma (NHL) that usually expresses B‐cell lineage antigens including CD20.[1] CD20 is a 33‐37 kDa, non‐glycosylated phosphoprotein expressed on the surface of almost all normal and malignant mature B cells that can be directly targeted by monoclonal antibodies like Rituximab.[2]

CD20 negative (CD20^‐^) DLBCL is rare and includes sub‐types such as primary effusion lymphoma, plasmablastic lymphoma, ALK^+^ large B‐cell lymphoma (ALK^+^LBCL), and large B‐cell lymphoma arising in HHV8^+^ multicentric Castleman disease (HHV8^+^ MCD).[3] Association with HIV, HHV‐8, and EBV has been noted in most subtypes with the exception of ALK^+^LBCL.[4] Survival except with HHV8^+^ MCD, is reported to be worse than DLBCL‐NOS (not otherwise specified).[3]

We report a case of a 90‐year‐old woman with background history of metastatic gall bladder adenocarcinoma who was diagnosed with CD20^‐^ DLBCL. Prior to initiating therapy for adenocarcinoma, investigation of dysphagia identified a lesion at the base of tongue. Morphological features on biopsy of the mass showed confluent areas of centroblastic cells with vesicular nuclei and prominent nucleoli, admixed with centrocytic cells. Immunohistochemical staining was positive for PAX5, CD79a, BCL2, BCL6, CD10, and MUM1 while CD20, CD30, ALK‐1, and EBER‐ISH were negative. Ki‐67 staining was positive in 80% of the lymphoid population (Figure 1). Immunophenotyping using flow cytometry confirmed a clonal population of dim kappa light chain restricted B‐lymphocytes expressing CD19 and CD22 with abnormal loss of CD20.

Due to her age and frailty, she was palliated with low dose gemcitabine that was ceased when local progression of gall bladder malignancy occurred. The patient received end of life care and died soon afterward.

1

**FIGURE 1:**
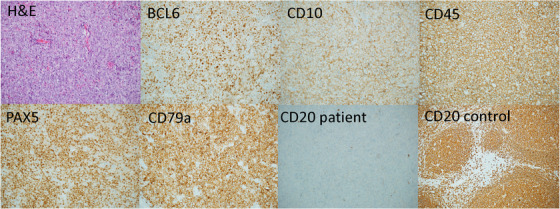
Haematoxylin and eosin staining shows features consistent with DLBCL, and immunohistochemical stains (×100 magnification) show positive staining with CD45, CD10, CD79a, PAX5, and Bcl6. CD20 staining was absent on the patient sample while control tissue showed positive staining

## 2 SOMATIC MUTATIONS IDENTIFIED BY NEXT‐GENERATION SEQUENCING

We extracted genomic DNA and performed next generation sequencing (NGS) on fresh frozen diagnostic tissue using a customized capture library (SureSelectXT Target Enrichment System, Aqilent Technologies, Cat# G9707B) targeting genes involved in lymphomagenesis. The purified libraries were sequenced on the Illumina NextSeq500 platform in Australian Genome Research Facility, Melbourne, Australia.

We identified the following mutated genes in our patient: *TANK and TNFRSF11a/RANK, EZH2, HIST1H1C, HIST1H1E, KMT2B, PRDM2, NOTCH2, ATM, BCOR, CIITA, SPEN, TCF3*, and *TYK2 genes, DTX1 ubiquitin ligase* (three individual mutations located in WWE1 domain), *ADAMTS5, SYNE1, ZFHX4, XPC, FCGR3A*, and *BRAF‐V600E*.

Notably, mutations in MS4A1 (which encodes CD20) that inhibit binding of anti‐CD20 monoclonal antibodies, were not detected.

Mutations previously reported in DLBCL are TANK and TNFRSF11a/RANK (positive regulators of nuclear factor (NF)‐κB pathway), EZH2, HIST1H1C, and HIST1H1E (chromatin modulating proteins), KMT2B/MLL2 (encodes histone methyltransferase), DTX1 (WWE1 domain), CIITA, FCGR3A, and ATM (Ataxia‐telangiectasia). Some of these mutations, for example, EZH2, HIST1H1C, and HIST1H1E, KMT2B/MLL2, NOTCH2, CIITA, TANK, and ATM are also reported in other lymphoproliferative disorders.

Mutations not associated with DLBCL but reported in other lymphoproliferative disorders were also identified. XPC and TANK have been identified in Hodgkin Lymphoma (HL); BCOR in T‐prolymphocytic leukemia (T‐PLL), Chronic lymphocytic leukemia (CLL), diffuse splenic lymphoma of the red pulp and extranodal NK/T‐cells lymphoma, nasal type; TYK2 in anaplastic large cell lymphoma (ALCL); TCF3 in Burkitt lymphoma (BL) and B‐lymphoblastic lymphoma (B‐LBL); ZFHX4 in B‐LBL, and BRAF‐V600 in Hairy cell leukemia (HCL). PRDM2 mutations have been associated with lymphomagenesis but not previously described in DLBCL cases.

Novel mutations of unknown significance not described previously in lymphoproliferative disorders included ADAMTS5 and SYNE1.

## 3 MUTATIONS WITH PROGNOSTIC RELEVANCE

NOTCH2 is recurrently mutated across a spectrum of B cell malignancies including follicular lymphoma (FL), mantle cell lymphoma (MCL), splenic marginal zone lymphoma (SMZL), CLL, BL, and HL. Altered NOTCH pathway signaling in DLBCL and other lymphomas is associated with shorter PFS and OS.[5] Mutated CIITA causes loss of MHC class II expression leading to immune escape and treatment resistance. It has been reported in primary mediastinal B cell lymphoma that low expression is an independent predictor of poor survival in DLBCL.[6] ATM gene has a role in DNA repair and somatic mutations of this gene are commonly found in lymphoid malignancies including T‐PLL, MCL, and CLL. These are associated with adverse prognosis and short PFS.[7] A germline mutation in ATM with predisposition to DLBCL has also been described.[8]

Other mutations of prognostic significance include those of NF‐κB pathway that is responsible for the regulation of multiple cellular processes. Constitutive activation of NF‐kB is particularly found in the activated B‐cell like subtype of DLBCL. DTX1 ubiquitin ligase mutations (WWE1 domain) are reported as a novel negative predictor of survival in DLBCL [9]. KMT2B/MLL2 is mutated more frequently in FL as compared to DLBCL, and MLL2 protein overexpression is proposed as a prognostic marker in gastrointestinal DLBCL.[10]

## 4 POTENTIALLY TARGETABLE MUTATIONS

While the mutations identified in our cohort require study in larger cohorts, this index case nevertheless provides information on targetable mutations that may provide potential novel therapeutic options given the lack of utility of anti‐CD20 monoclonal antibodies in this subtype of lymphoma.

Development of γ‐secretase inhibitor targeting NOTCH signaling (used in combination with chemotherapy) has emerged as a therapeutic strategy for high risk CLL patients [11] and may have a potential therapeutic role in DLBCL.

EZH2 is a histone methyltransferase and gain‐of‐function somatic mutations are detected in germinal center B‐cell derived DLBCL. The oral EZH2 inhibitor Tazemetostat has shown positive results as a single agent in a phase 2 clinical trial for relapsed or refractory FL[12] and a phase 2 study investigating clinical activity in DLBCL is underway (NCT01897571).

TYK2 is a member of Janus kinase family and activates STAT signaling. TYK2 inhibitor has shown activity against ALCL in a preclinical model[13] and could be a potential novel drug target. BRAF‐V600E is a driver mutation in HCL and is targetable with Vemurafenib[14] showing high efficacy.

As far as we are aware, this is the first report on the mutational profile of CD20‐ DLBCL. We have identified a number of targetable mutations that may provide alternative treatment options. Given the rarity of this subtype of lymphoma, international collaboration to genotype larger cohorts and identify recurrent mutations is required. Genotyping of individual cases in a personalised medicine approach may improve outcomes associated with this poor risk disease.

## CONFLICT OF INTEREST

The authors declare no conflict of interest.
